# The effect of COVID-19 pandemic on final year dental students’ self-confidence level in performing clinical procedures

**DOI:** 10.1371/journal.pone.0257359

**Published:** 2021-10-14

**Authors:** Jugoslav Ilić, Katarina Radović, Tatjana Savić-Stanković, Aleksandra Popovac, Vesna Miletić, Aleksandra Milić Lemić

**Affiliations:** 1 Department of Restorative Dentistry and Endodontics, School of Dental Medicine University of Belgrade, Belgrade, Serbia; 2 Department of Prosthetic Dentistry, School of Dental Medicine University of Belgrade, Belgrade, Serbia; 3 School of Dentistry, Faculty of Medicine and Health, The University of Sydney, Surry Hills, New South Wales, Australia; University of Queensland, AUSTRALIA

## Abstract

**Background:**

The outbreak of COVID-19 pandemic in 2020 has dramatically changed teaching approach in dental schools due to the switch to distance learning and the lack of practice training in direct contact with patients with possible impact on clinical skills of students. The aim of the study was to assess the level of the 2020 final year dental students’ self-confidence in performing different dental procedures through specially designed questionnaire and compare it to self-confidence of the 2019 final year students.

**Materials and methods:**

An anonymous questionnaire consisting of 40 questions regarding self-confidence level in performing 40 different dental procedures and based on five points Likert-like scale was distributed during November 2020 to final year dental students whose studies were disrupted by the COVID-19 pandemic lockdown in one semester. The study group comprised their answers. The comparison was done with the control group that consisted of students’ answers on the same questionnaire from previous 2019 year conducted as a part of regular internal educational evaluation.

**Results:**

Response rate was 74.2% in study group and 89.3% in control group. Mean level of self-confidence reported by 115 students in study group was significantly lower than that reported by 100 students in control group (3.28±1.08 vs. 3.58±0.88, respectively) and the distribution of self-confidence scores were different in observed groups. Graduates from study group felt less confident than those from control in 8 clinical skills.

**Conclusion:**

Abrupt changes in teaching modalities caused by COVID-19 pandemic had significant impact on final year dental students’ self-confidence indicating additional educational needs in postgraduate period.

## Introduction

Constant knowledge growth in the area of biomedical sciences continually influences modern dental education. Hence, dental schools and universities are under constant demand for providing education that follows science and practice and involves solving real-life situations, interpersonal interactions with patients, practice-based learning and gaining clinical experience [1]. Listed above are also of paramount importance in current dental school curricula, since it is widely recognized that an effective training of dental students improves both the performance and success of clinical procedures performed later by them [2]. Further, the essential dental professional clinical skills include: diagnosis and treatment planning, preventative measures and patient treatment and rehabilitation [3]. In order to prepare students for their future professional life, teachers of dental schools and universities foster their readiness and confidence for direct patient care, boosting their psychomotor skills, expanding knowledge in terms of diagnosis, treatment options and planning thus upgrading their clinical competences [4]. Accordingly, it is upon universities and dental schools to create academic environment, provide materials, equipment and facilities that best suit students to attain competencies for their future professional life and consequent benefit for the patients.

Variety of pedagogical models are currently available in dental teaching, such as traditional amphitheatre lectures, problem-based learning and case-based learning, clinical seminars, active group discussions, the use of contemporary communication technologies, the development of portfolio, self-assessment and formative assessment [5–7]. Although different teaching methods and educational standards may influence the graduates’ confidence and competence [8], modern dental teaching managed to create ‘fully qualified beginners’ [9], prepared for life learning process with possession of adequate knowledge together with cognitive, interpersonal and clinical skills. Furthermore, important aspect of achieving competency is the self-confidence and learning ability [3]. Apart from the training environment, the students’ attitude and self-confidence in handling a specific procedure are crucial for the final outcome of the learning process [2]. Thus, self-confidence is an extent of dental graduate’s competence. The importance of achieving self-confidence is an asset in enhancing the competencies, whereas it is crucial to teach a student to self-evaluate to avoid overconfidence [10].

The outbreak of COVID-19 pandemic in spring of year 2020 has dramatically changed both teaching and learning methods in dental school [11] with strong impact on the knowledge asset of current dental students. It has been claimed that COVID-19 pandemic massively affected clinical dental practice and dental education [12]. The lockdown declared in most countries as an attempt to prevent virus spread, forced universities, dental schools and affiliated hospitals to close their premises for students, teachers and patients while traditional teaching methods have been suspended. The teaching concept of our school before the COVID-19 pandemic was according to curriculum that comprises courses of one or two semesters, that are distributed over 6 years studies and consists of “face-to-face” theoretical lectures and practical classes (preclinical or clinical). In the spring semester 2020, due to total lockdown declared in our country, sustaining level of teaching activities in our dental school was transferred to on-line learning platform (Google Classroom^®^), with dominant pre-recorded theoretical lectures, case presentations and electronic learning tutorials. Similarly to vast majority of worldwide dental schools [13, 14], dental clinics of our University from that moment were available only for treatment of dental emergencies, and all appointments that students scheduled were postponed since practical courses ceased. Practical courses were introduced back in October 2020 at significantly reduced level and with rigorous preventive measures, while theoretical lectures remained online. There was not synchronized national strategy for system of higher dental education during COVID-19 pandemic, since curricula of all dental schools differ significantly. Therefore, each school has had its own modification of educational process, although the common to all are the cease of practical courses and introduction of some kind of on-line learning. Noteworthy, the positive impact of transition to on-line teaching is reported as improvement of learning efficiency and performances by adopting online learning strategies [15]. Although our school rapidly adopted on-line lecturing and teachers efficiently shifted to distant teaching, students were restrained from direct contact with patients. That affected their ability to apply gained knowledge, develop critical thinking in clinical setting and build experience. Undoubtedly, the switch to distance learning and the lack of practical training during the semester could leave students without proper confidence in performing dental procedures. Many investigations have been performed so far concerning different aspects of the impact of COVID-19 pandemic on dental education [16, 17]. However, as far as we are aware none of the published studies compared the students’ educational results during COVID-19 pandemic with those gained from previous years although it would be informative from at outcome point of view [18].

Therefore, we conducted the comparative study with the aims: i—to assess final year dental students’ self-confidence in performing different dental procedures after the COVID-19 lockdown and consequential changes in teaching methods and academic activities during one semester in spring of 2020, and ii—to compare obtained results for dental graduates’ self-confidence in performing different dental procedures after the COVID-19 with the self-confidence rates of students from the previous year. The null hypothesis was: self-confidence of final year dental students in performing dental procedures after one semester of online lectures and cease of clinical work is not significantly different from those who attended courses on regular basis.

## Materials and methods

### Ethical approval

Ethical approval was obtained from the University of Belgrade Dental School Ethics Committee (36/25, date 23.10.2020).

### Study design and study participants

The survey included the final, sixth year dental graduating students who were fifth year of studies during the COVID-19 lockdown in first wave of pandemic and encountered on line learning and disruptions in practical academic activities. They comprised the study group (SG_2020) with a sample size of 115.

The level of self-confidence of the 2020 final year dental students ’ in performing different dental procedures was assessed through specially designed on line questionnaire. A set of 10 questions was chosen from Conservative Dentistry, Prosthodontics, Oral Surgery, and Periodontology making total of 40 questions corresponding to 40 different dental procedures related to various fields of dentistry. Moreover, procedures from Conservative Dentistry were further divided into two sets of five questions assigned to Restorative Dentistry and Endodontics, and similarly Prosthodontic procedures were spread in two sets of five for Removable and Fixed Prosthodontics. The procedures included in the questionnaire are derived from the competencies students should have on completion of particular courses as stated in curriculum of our School.

A self confidence level was assigned by the student participant according to the five-point scale ranging: 1 (no self-confidence), 2 (little confidence), 3 (moderate confidence), 4 (confident), and 5 (very confident).

The inviting cover letter with informed consent and the link to the on-line questionnaire were distributed to all students of the final academic year via personal e-mails. In such way, they were issued with an invitation to participate by completing the on-line questionnaire (available in S1 and S2 Appendices). Furthermore, the purpose of the investigation was outlined and thorough explanation that the participation is completely voluntary and entirely anonymous was given. Also, it was noted that by completing the on-line questionnaire it would be considered that the respondents had given the written consent for participating and utilization of the gained data for statistical analyses within the current investigation. In addition, it was made clear that students’ decision to participate or not, as well as the given answers would have no impact on their current studying status at the Dental School. The responders to this invitation were allocated to SG_2020 group.

The results of the previous final year dental students’ self-evaluation survey were allocated to the control group (CG_2019) of the investigation. The CG_2019 included answers from the data base of the 100 students rating their self confidence level in performing dental procedures according to the described questionnaire. This kind of questionnaire is regularly used in our school at the end of each school year for the purpose of internal self-evaluation of educational process, where the final year dental students self-rate their clinical confidence among the other questions. The survey including this questionnaire is a part of the internal quality control and monitoring.

### Data analysis

The questionnaire was assessed before starting the study for reliability and internal consistency using previously collected data included in CG_2019 with Cronbach’s α. Descriptive statistics of the reported self-confidence levels are presented by mean values, standard deviation and median values. Proportions (in %) were used for the sample structure and response rate. The analysis of these data was done using z test since the size of sample was adequate (n>30). Self–confidence scores were analyzed at the level of whole sample as well as observing single questions and question groups according to dental fields. Significance of differences in self-confidence scores for the whole sample was evaluated using t test and inter-area comparison was done using one-way analysis of variance (ANOVA) with Tukey post hoc test for pairwise comparisons. Distributions of the scores were compared with Kolmogorov-Smirnov test, having in mind that the study group and control contained different number of examinees. When comparing skills within particular dental fields, self-confidence scores missed normal distribution and the analysis at this level was conducted with Kruskal-Wallis test. Significance level for all tests was set at α = 0.05. The software used in analysis were Microsoft Excel^®^ and SigmaPlot^®^ 12.0.

## Results

The reliability level of the questionnaire was satisfactory with Cronbach’s α of 0.951. Response rate in the study group was 74.2% (115 respondents out of 155 students attending final year), while in the control group it was 89.3% (100 out of 112 students). Total number of answers was 4600 in the study group and 3999 in the control group (one question was omitted by a respondent). The difference in response rate between these two groups was significant (z = 2.919, p = 0.004). Female responders were dominant in both groups (80% in study group and 71% in control). There was no difference in gender structure between the groups (z = 1.377, p = 0.168).

**Fig 1:**
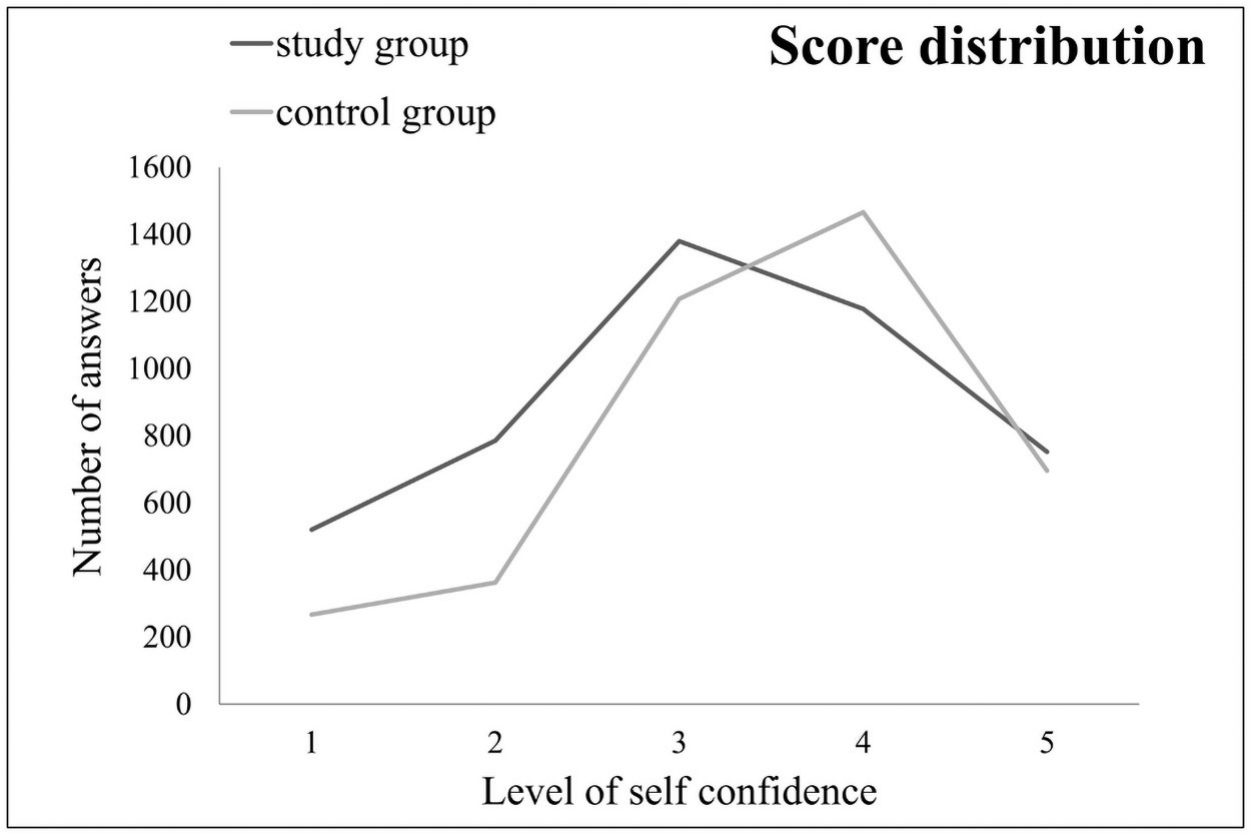
The distributions of self-confidence scores assigned by students in the study and the control group. Study group consisted of final year students that were on distant learning regime from 15^th^ March 2020 to the end of 10^th^ semester (15^th^ Jun 2020) due to COVID-19 pandemic. Control group consisted of final year students from previous year that were on standard teaching protocol according to syllabus. Significant difference between these distributions was detected (Kolmogorov-Smirnov test, p<0.001).

Mean level of self-confidence reported by students in study group was 3.28±1.08, significantly lower than in control group (3.58±0.88; t = -13.987, p<0.001). Likewise, the distribution of self-confidence scores are different in observed groups (D = 0.126, p<0.001) as shown in Fig 1. The mean values of self-confidence levels for specific dental field differ mutually in study and control group, except for the field of Fixed Prosthodontics (Table 1).

**Table 1:** Mean values of students’ self-confidence for specific dental fields in the study group in comparison to control.

Dental field	Study group	Control group	p value
	Mean± SD	Mean± SD	
Restorative dentistry	3.88±0.90	4.29±0.75	<0.001
Endodontics	3.33±1.06	3.68±0.80	<0.001
Removable prosthodontics	3.45±1.07	3.80±0.86	<0.001
Fixed prosthodontics	3.23±1.19	3.31±0.96	0.981
Periodontology	2.93±1.13	3.38±0.95	<0.001
Oral surgery	2.86±1.15	3.04±0.98	0.002

Five-point scale ranging: 1- no self-confidence, 2- little confidence, 3- moderate confidence, 4- confident, and 5- very confident. Scores were compared using one way ANOVA; post hoc: Tukey test. SD = Standard deviation.

**Fig 2:**
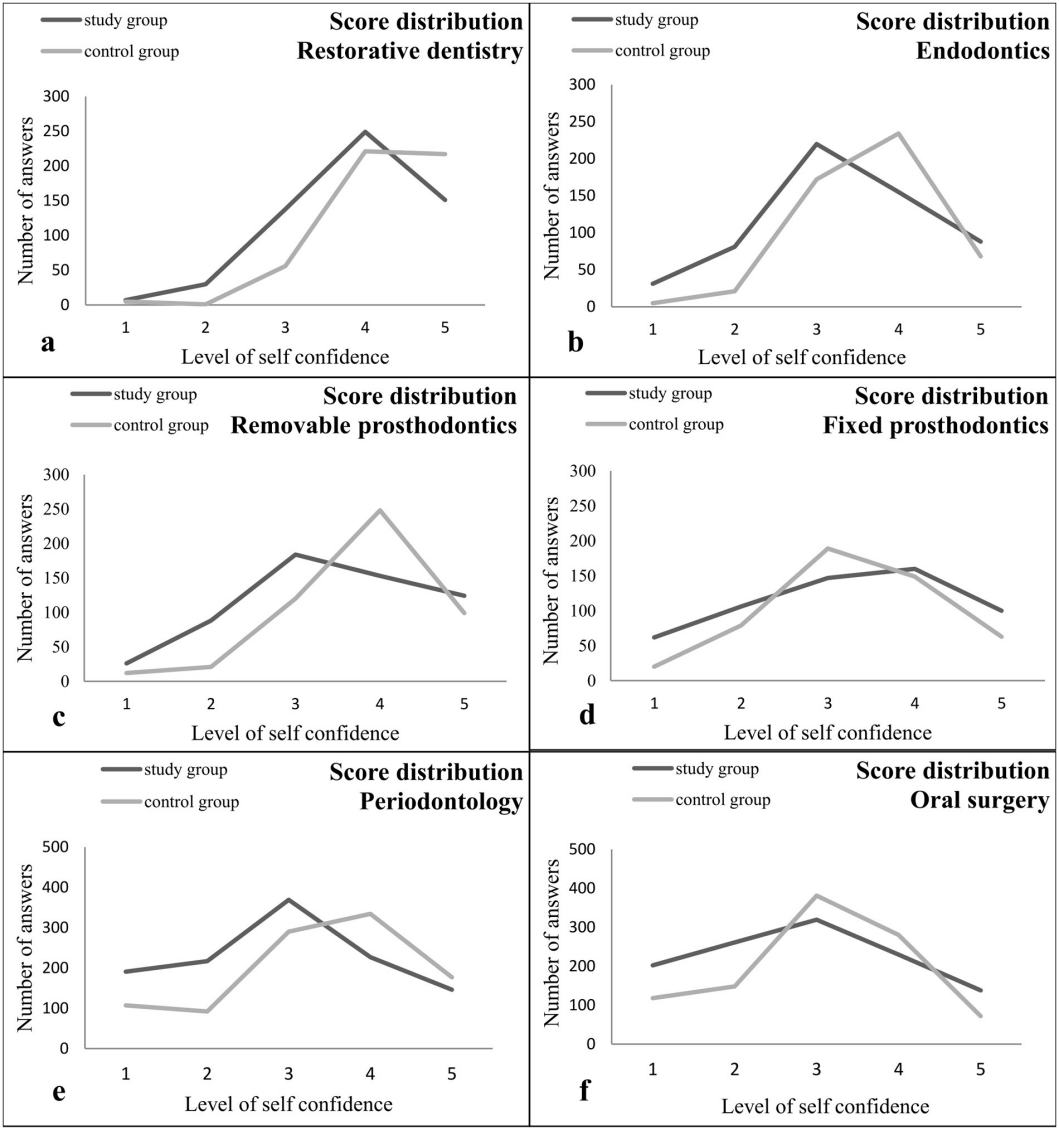
The distributions of self-confidence scores for procedures in restorative dentistry (a), Endodontics (b), Mobile prosthodontics (c), Fixed prosthodontics (d), Periodontology (e) and Oral surgery (f) assigned by students in the study and the control group. Study group consisted of final year students that were on distant learning regime from 15^th^ March 2020 to the end of 10^th^ semester (15^th^ Jun 2020) due to COVID-19 pandemic. Control group consisted of final year students from previous year that were on standard teaching protocol according to syllabus. Significant difference between these distributions was detected (Kolmogorov-Smirnov test, p<0.001).

Analysis of the responses on particular questions considering skills in the field of Restorative Dentistry showed statistical difference (p<0.05) in perceived self confidence levels between the control and the study group for two skills: ‘Maintaining of records in Restorative dentistry’, and ‘Caries diagnostics and planning of therapy’ (Table 2). No significant differences were identified between different skills within group, although the procedure ‘Clinical examination in Restorative Dentistry’ was with the highest self confidence level in both control group and study group. There is significant difference in distributions of scores for restorative procedures between study and control group (D = 0.18, p<0.001, Fig 2a).

**Table 2:** Self-confidence levels assigned by students in study and control group for procedures in restorative dentistry.

Restorative procedure	Study group		Control group	
	mean±SD	median	mean±SD	median
1.Clinical examination in restorative dentistry	4.02±0.90	4	4.40±0.72	5
2.Maintaining of dental records in restorative dentistry*	3.68±1.04	4	4.27±0.78	4
3.Caries diagnostics and planning of therapy*	3.82±0.87	4	4.24±0.74	4
4.Cavity preparation	3.91±0.83	4	4.28±0.77	4
5.Placement of all types of tooth restorations	3.89±0.86	4	4.25±0.73	4

Five-point scale ranging: 1- no self-confidence, 2- little confidence, 3- moderate confidence, 4- confident, and 5- very confident. Scores were compared using Kruskal Wallis test with Dunn’s post hoc (p<0.05). SD = Standard deviation.

*Significant difference in self-confidence level for the procedure between study and control group.

Significant difference was noticed in score distribution when performing Endodontic procedures (D = 0.184, p<0.001; Fig 2b). However, no significant differences were observed when particular questions were compared between the groups as well as within the group (Table 3).

**Table 3:** Self-confidence levels assigned by students in study and control group for procedures in endodontics.

Endodontic procedure	Study group		Control group	
	mean± SD	median	mean± SD	median
1.Pulpal diagnosis	3.35±0.98	3	3.69±0.76	4
2.Diagnosis of apical periodontitis	3.30±1.02	3	3.57±0.88	4
3.Endodontic therapy- root canal preparation	3.33±1.06	3	3.71±0.80	4
4.Endodontic therapy-irrigation and medication	3.23±1.08	3	3.64±0.77	4
5.Endodontic therapy- root canal obturation	3.43±1.18	3	3.78±0.77	4

Five-point scale ranging: 1- no self-confidence, 2- little confidence, 3- moderate confidence, 4- confident, and 5- very confident. SD = Standard deviation.

No significant differences were detected considering self confidence levels for particular procedures within and between the groups (Kruskal Wallis test, Dunn’s post hoc: p>0.05).

The analysis of questions in the field of Removable Prosthodontics showed that perceived level of self-confidence for ‘Establishing of intermaxillary relationships’ was significantly lower in the study group than in the control (p<0.05). Also, this procedure was with the least self confidence level within both groups. Significant differences were detected in four comparisons of procedures within the study group and two comparisons in the control (Table 4). Consequently, significant difference in distributions of scores for Removable Prosthodontics skills was revealed between groups (D = 0.21, p<0.001, Fig 2c).

**Table 4:** Self-confidence levels assigned by students in study and control group for procedures in removable prosthodontics.

Removable prosthodontics procedure	Study group		Control group	
	mean±SD	median	mean±SD	median
1.Anatomic and situational impressions	3.75±1.06	4	4.09±0.83	4
2.Functional impression for removables and complete dentures	3.50±1.04	3	3.66±0.78	4
3.Establishing of intermaxillary relationship*	2.82±1.09	3^1,2,4,5^	3.5±0.93	4^1,5^
4.Teeth set up try in	3.45±1.13	3	3.74±0.87	4
5.Delivery of dentures	3.74±1.05	4	4.02±0.89	4

*Significant difference in self-confidence level for the procedure between the study and the control group.

Superscript: Significant difference within the group; in comparison to procedures numbered in superscript. The number of procedure given in superscript is the same as in the first column of the table.

Five-point scale ranging: 1- no self-confidence, 2- little confidence, 3- moderate confidence, 4- confident, and 5- very confident. Scores were compared using Kruskal Wallis test with Dunn’s post hoc (p<0.05). SD = Standard deviation.

Perceived levels of self-confidence on questions related to Fixed Prosthodontics did not differ between the study group and the control. However, there were differences in four comparisons of skills within the study group and three comparisons in the control group (Table 5). The least confidence was reported for ‘Tooth preparation in Fixed Prosthodontics’ in both groups. Students in both groups were most self-confident in ‘Cementing of fixed partial denture’. Significant differences in score distribution between the study group and the control existed at accepted level of significance (Fig 2d).

**Table 5:** Self confidence levels assigned by students in study and control group for procedures in fixed prosthodontics.

Fixed prosthodontics procedure	Study group		Control group	
	mean±SD	median	mean±SD	median
1.Root canal preparation for casted post	3.05±1.18	3	3.23±0.94	3
2.Tooth preparation in fixed prosthetics	2.63±1.22	3^3,4^	2.69±1.00	3^3,4,5^
3.Impressions in fixed prosthetics	3.43±1.19	3	3.47±0.90	3
4.Try-in of ceramic fused to metal reconstructions. Rearticulation	3.36±1.21	4	3.53±0.92	4
5.Cementing of fixed prosthetic	3.65±1.17	4^1,2^	3.64±1.01	4

Superscript: Significant difference within the group; in comparison to procedures numbered in superscript. The number of procedure given in superscript is the same as in the first column of the table.

Five-point scale ranging: 1- no self-confidence, 2- little confidence, 3- moderate confidence, 4- confident, and 5- very confident. Scores were compared using Kruskal Wallis test with Dunn’s post hoc (p<0.05). SD = Standard deviation.

Significant difference in the level of self-confidence between the study and the control group was detected in even five questions in the field of Periodontology (Table 6). Differences within the study group were noticed in 18 skill comparisons and in 22 comparisons in the control group (Table 6). The least confidence was reported for procedure ‘Surgical elimination of periodontal pockets’ in both groups. The procedure that students felt most confident in both groups was ‘Causal therapy of periodontal disease’. However, significant difference in the level of perceived self-confidence for this procedure was noticed between the study group and the control. Overall distribution of scores was significantly different between study group and control (D = 0.187, p<0.001; Fig 2e).

**Table 6:** Self-confidence levels assigned by students in study and control group for procedures in periodontology.

Periodontal procedure	Study group		Control group	
	mean±SD	median	mean±SD	median
1. Diagnosis of periodontal pathology *	3.17±1.02	3	3.77±0.80	4
2. Causal therapy of periodontal disease*	3.61±1.04	4	4.32±0.72	4^6,8,9,10^
3. Diagnosis of mucogingival anomalies*	3.10±0.99	3	3.71±0.83	4
4. Correction of mucogingival anomalies.	2.23±1.19	2^1,2,3,7,8,9,10^	2.31±1.15	2^1,2,3,6,7,8,9^
5. Surgical elimination of periodontal pockets.	1.82±1.23	1^1,2,3,7,8,9,10^	1.97±1.12	2^1,2,3,6,7,8,9^
6. Diagnosis and elimination of traumatic dental contacts*	2.58±1.23	2^2,5,7,8^	3.46±0.98	3.5
7. Evaluation of causal therapy success*	3.25±1.05	3	4.09±0.78	4^6,10^
8. Recognition of risk factors for periodontal disease	3.39±1.05	3	3.62±0.85	4
9. Swab taking.	3.11±1.31	3	3.55±1.02	4
10. Elimination of local irritations. Local medication	3.03±1.20	3	3.02±1.21	3^1,3^

*Significant difference in self-confidence level for the procedure between the study and the control group

Superscript: Significant difference within the group; in comparison to procedures numbered in superscript. The number of procedure given in superscript is the same as in the first column of the table.

Five-point scale ranging: 1- no self-confidence, 2- little confidence, 3- moderate confidence, 4- confident, and 5- very confident. Scores were compared using Kruskal Wallis test with Dunn’s post hoc (p<0.05). SD = Standard deviation.

Significant differences were not observed when comparing particular skills in Oral surgery between groups. Significant differences were noticed in 20 comparisons of skills within the study group, and in 14 skill comparisons in the control group (Table 7). Procedure that students felt the least confident in was ‘Surgical extractions of fractured radices and partially erupted teeth’, both in the study group and the control. Procedures with most self-confidence were: ‘Terminal and block anaesthesia in oral tissues…’ and ‘Extraction of erupted teeth and wound management’ in both groups, while ‘Haemostasis in tooth extractions and minor oral surgery’ was with most self-confidence exclusively in the study group. The distribution of scores differed significantly (D = 0.136, Fig 2f).

**Table 7:** Self-confidence levels assigned by students in study and control group for procedures in oral surgery.

Procedure in Oral surgery	Study group		Control group	
	mean±SD	median	mean±SD	median
1. Administering of local anesthesia in oral tissues.	3.70±1.00	4^3,4,5,6,7,10^	3.83±1.09	4^3,4,5,6,8,9,10^
2. Extraction of erupted teeth and wound management.	3.78±1.01	4^3,4,6,7,9,10^	3.85±0.82	4^3,4^
3. Tooth extractions with separation of roots.	2.38±1.25	2	2.41±1.18	2^9^
4.Surgical extractions of fractured radices	2.02±1.28	1^5,6,7,8,10^	2.05±1.08	2^5,6,8,9^
5.Recognition of indications for extraction of impacted teeth.	2.70±1.18	3	2.88±1.03	3
6.Diagnosis and therapy of acute and chronic dental infections	2.74±1.15	3	2.84±0.92	3
7. Tooth extraction and minor oral surgery in medically compromised patients.	2.77±1.09	3	3.32±0.91	3
8.Haemostasis in tooth extractions and minor oral surgery	3.19±1.20	3^3,4,9^	2.91±1.08	3
9.Diagnosis and conservative therapy of oroantral communications and fistulas.	2.57±1.17	3	3.1±0.89	3
10.Diagnosis of periapical lesions and cysts in jaws.	2.77±1.16	3	3.18±0.83	3

*Significant difference in self-confidence level for the procedure between the study and the control group

Superscript: Significant difference within the group; in comparison to procedures numbered in superscript. The number of procedure given in superscript is the same as in the first column of the table.

Five-point scale ranging: 1- no self-confidence, 2- little confidence, 3- moderate confidence, 4- confident, and 5- very confident. Scores were compared using Kruskal Wallis test with Dunn’s post hoc (p<0.05). SD: Standard deviation.

## Discussion

Self-confidence level in performing 40 different clinical dental procedures of the 2020 final year dental students was the main outcome of this study. We compared it with the self-confidence values obtained from the previous year graduates where impact of COVID-19 pandemic lockdown on teaching process did not exist. As far as we are aware this is the first study to consider impact of changes in educational process due to the COVID-19 pandemic on the knowledge asset based on comparison of students’ self confidence in pre-pandemic and pandemic period.

The existing questionnaire was used in order to collect the set of data comparable to CG_2019 and to avoid time consuming construction of new instrument that would delay data acquisition and cause inaccurate responses by the participants due to time distance. In support of this were high level of reliability indicated by satisfactory level of Cronbach’s α [19] and the fact respondents did not report any problem with reading, instructions, item clarity and response categories regarding this instrument.

The response rate in control population investigated in 2019 was very high. Since the same was expected in target population the preliminary estimation of required sample size was not conducted. Indeed, the achieved response of 74.2% indicated that majority of the whole population to be examined was presented in the sample. Additionally, high response rate was in favour of reducing bias of not representative data. Students scored self-confidence level using five-level Likert like format (from 1-no self-confidence to 5- very confident). Since the response format in the questionnaire consisted of five categories, according to Harpe [20], we treated these as continuous variables and reported means and standard deviations for each item as well as at the level of whole groups and in particular dental specialities. For the sake of completeness, we reported median value of reported self-confidence on each question. The above mentioned ‘intervalistic’ approach enabled the use of more powerful parametric mode of analysis. For additional support and findings, we analysed distributions and compared each item in non-parametric mode. As expected, null hypothesis was rejected since significant differences in self-confidence scores and distribution were found. It can be claimed that 2020 final year dental students experienced notable influence of lack of direct contact with patients and shift to distance learning due to COVID-19 lockdown on their self-confidence in performing routine dental procedures. This is in concordance with reported perception of students and educational staff from University of Otago that the impact of suspension of clinical activities would be extreme [12].

Final year dental students from study group felt less confident than those from control in 8 clinical skills and significant change in self-confidence was not seen only in performing procedures from the field of Fixed Prosthodonitcs. Self-confidence of 2020 graduating students for other dental disciplines observed in this study was with statistically significant difference compared to the self-confidence of the final year dental students from the previous year. No doubt, described may be influenced by the lack of sufficient clinical practice due to lockdown. Among all of the 40 investigated clinical procedures, trainings of the 20 that belong to Fixed Prosthodontics, Endodontics and Periodontology are positioned at IX and X semester of dental curriculum in our school, while X semester was heavily affected with the lockdown. It is evident that our 2020 year graduating dental students lost most precious time needed for establishing and boosting practical skills concerning mentioned dental disciplines.

As shown, the observed scores of self-confidences in discipline of Fixed Prosthodontics were low both in study group and control and they did not differ mutually. Although the last mentioned was unexpected it may be attributed to the fact that overall level of clinical experience students gain in this area was low. These procedures are complex and require comprehensive knowledge and manual skills, but nonetheless the results highlighted the main drawback of the current curriculum concerning Fixed Prosthodontics courses.

The highest values for self-confidence final year dental students scored for procedures involving Restorative Dentistry, Removable Prosthodontics and simpler procedures from Oral Surgery. These are the clinical courses that observed students finished during the year preceding the pandemic. No doubt, they have lost time for refining these skills during the COVID-19 lockdown and, therefore, their scores are significantly different from the final year dental students of the previous year. Similarly, students in the study from Jordan found Conservative Dentistry as most negatively affected by the COVID-19 lockdown and the authors stated it was comprehendible since the bulk of students’ clinical exposure during their final years revolved around Restorative Dentistry [18]. We have included in the questionnaire a significant number of questions based on more complex procedures from Oral Surgery since they are listed as competences in curriculum of our school. Students scored them with lower grades in both study and control group resulting in lower overall grade in the field of Oral Surgery compared to Restorative Dentistry and Removable Prosthodontics. This could be indicative for need of corrective measures in curriculum in this area such as excluding some of competencies or change in teaching modalities for them. Our graduates reported higher scores for simpler Oral Surgery procedures and lower for complex ones and this is in accordance with the findings for graduates from Cardiff and Cork [21]. Noteworthy, our students felt confident in performing most of the procedures from Removable Prosthodontics, except for the ‘Establishing of intermaxillary relationship’ and the same was found for the students of Porto [22].

Similarly to Oral Surgery, analysis of self-confidence levels for skills in Periodontology, showed that our students in both groups assigned low scores for complex periodontal interventions like “Correction of muco-gingival anomalies” and “Surgical elimination of periodontal pockets”. Over and above, these are the procedures that were rarely performed by students and, as in a majority of the dental schools, students of the 5th year mostly observes surgical procedures performed by faculty staff [23] and it was confirmed that the most important factor affecting students’ preparedness was indeed their clinical exposure [24].

As anticipated, self–confidence levels in Endodontics are lower for 2020 graduates but relatively uniform within the groups, suggesting that the impact of the cease of clinical practice is more profound for this area than the overall effect of change in teaching process. Accepting the statement that perception of confidence and competence when performing root canal treatment increases as the years progress [8], one could expect that self-confidence for complex endodontic procedures will increase.

The findings implicate that the decrease in self-confidence levels is not the result of solely cease of teaching in clinical setting, but rather of the combination of the impact of pandemic on teaching process and the influence of other effects such as curricular factors (location and duration of courses), students’ interests and efforts of teaching staff. Namely, the skills that were gained during the courses that completely finished before the outbreak of COVID 19 were also affected. Also, the self-confidence was affected not only by lowering the perceived level but also by the change in the distribution of scores in observed groups. Disturbed distributions are probably the effect of differences in clinical load needed for proper adoption of different skills.

Overall, the results of this study revealed that 2020 final year dental students beside their lower self-confidence will start their professional career with the lack of number of clinical skills. A study from Germany also highlighted that students recognise their practical preparation not enough to make them feel comfortable to face the future profession [25]. Hence, it`s up to them to address their weaknesses in order to seek further training. Also, it is advisable for dental schools and their authorities to provide additional courses and workshops concerning the raised issue. In support of that is finding of Hattar et al. that nearly two-thirds of students included in their study preferred to be supervised following graduation [18]. What is encouraging is that there are procedures highlighted with higher scores regardless the abrupt change in teaching process and these are from the group of essential dental professional clinical skills [3] such as diagnosis and treatment planning. Although there are authors who stated that many students included in their studies did not feel well prepared for practical courses with online learning alone [26, 27], the positive aspects of on-line learning were reported both by student and teaching staff, such as higher motivation of students and easier participation resulting in suggested continuation of this educational model in order to improve future dental curricula [25]. Likewise, the sixth year students from Italy evaluated the COVID-19 pandemic modification of teaching method and defined it as significantly “fair” concerning the level of interactivity, level of knowledge and level of personal preparation [28]. Thus, it may be considered that implemented distance learning methods gave some results on students gaining comprehensive knowledge. In such a way they have created base for enriching and building their self-confidence in practical skills later.

The weakness of the study may be attributed to the fact that it is a questionnaire-based project and that final year dental students `self-confidence was evaluated instead of their competence, suggesting that subjective view was taken into analysis [29]. However, assuming that the competence and the perception of competence (self-confidence) are different [30] and implying that the level of confidence has implications for practice we implemented the self-confidence survey in our study. Another limitation is that the study was performed in single dental school. Although almost 50% of dental students in our country are attending the school included in this survey, the results cannot be extrapolated at the national level since the curricular differences are significant and the form of educational changes during lockdown was different. Therefore, students in other schools might respond differently [18], but we strongly believe that the strength of our study lies in an outcome point where educational results, manifested as self-confidence level, were compared with those results of previous year students.

The results of the research assess the final year dental students `subjective impression of self-confidence and indirectly the acquired knowledge and skills in the light of difficult teaching conditions due to the COVID-19 lockdown. The 2020 graduating students were the target group of the investigation, because they are on the threshold of the professional career and we anticipated that their self-confidence level would have great impact on their own career in the near future. Furthermore, it has been shown that students in the last year of their study expressed more serious interest in maintaining their practical skills [31]. The data obtained by this research indicate that these students, educated in specific conditions, do not feel safe in performing most of observed clinical procedures and they may have additional educational needs in post-graduation period. This work sheds light on implications regarding distance learning, raising issues in implementing new teaching modalities. It is advisable that dental schools re-evaluate their competency-based education, incorporate variations of distance learning permanently in their curricula, and invest in haptic technology to improve psychomotor skills and also in faculty training for teaching through technology [26] since it is now clear that the pandemic is long lasting [32] and the impacts of it are profound. Even after the contagion curve is flattened, it can be expected long lasting changes in dental clinic practice [33], as well as in dental education. Therefore, within the stated limitation, findings of this study could be the contribution in recognizing the significance of permanent implementation of educational experiences adopted during COVID-19 pandemic that could be useful in development of new teaching modalities in future dental education. Rising number of studies in this area is the warranty of global efforts to use different experiences in improving pedagogic approaches and preventing detrimental effects of sudden impacts on education process thus promoting collaboration between dental schools.

## Supporting information

S1 Dataset Students’ responses to the 2019 questionnaire. (XLS)

S2 Dataset Students’ responses to the 2020 questionnaire. (XLSX)

S1 Appendix Questionnaire and inviting cover letter with informed consent (in Serbian). (DOCX)

S2 Appendix Questionnaire and inviting cover letter with informed consent (in English). (DOCX)
