# Supplementary material for: The effect of COVID-19 pandemic on final year dental students’ self-confidence level in performing clinical procedures
Source: PLoS One. 2021 Oct 14;16(10):e0257359. doi: 10.1371/journal.pone.0257359 (PMC8516192; doi:10.1371/journal.pone.0257359)
Supplement: S1 Appendix — (DOCX) [file pone.0257359.s003.docx]

**Obaveštenje za učesnike anonimne ankete**

Poštovana koleginice, poštovani kolega

Pripadate generaciji studenata koja je deo svog stomatološkog obrazovanja dobila u posebnim uslovima pandemije oboljenja Covid 19. Verujemo da je ovo imalo uticaja ne samo na znanja i veštine koje ste usvojili, nego i na vašu samouverenost prilikom njihovog izvođenja. Zbog toga smo osmislili istraživanje u kome bi sakupili i analizirali Vaše stavove o sopstvenoj samouverenosti pri izvođenju stomatoloških procedura za koje bi trebalo da budete osposobljeni nakon ispita koje ste do sada položili. Rezultati ovog istraživanja bi pomogli u oblikovanju nastave ali i sagledavanju Vaših budućih potreba za edukacijom. Molimo Vas da uzmete učešće u ovom istraživanju tako što ćete popuniti anketu kojoj možete pristupiti putem [**ovog linka**](https://docs.google.com/forms/d/e/1FAIpQLSekeCKyYpNYDHj5olAufk-alznyhuC3kOhPnIxo2gx_CTn7VA/viewform?vc=0&c=0&w=1&flr=0). Popunjavanjem ove ankete dajete pristanak za učešće u istraživanju o stavu studenata VI godine studija o sopstvenom samopouzdanju pri izvođenju stomatoloških procedura. Takođe, dajete saglasnost da se dati odgovori koriste za statističku analizu u okviru pomenutog istraživanja. Anketa je anonimnai ne obuhvata davanjeVaših ličnih podataka, odnosno Vaši lični podaci neće biti dostupni istraživačima ni trećim licima. Popunjavanje ili nepopunjavanje ove ankete neće uticati na bilo koji način na Vaš status na Fakultetu.

Za istraživački tim,

Prof. dr Aleksandra Milić Lemić

Prof. dr Jugoslav Ilić

**Upitnik**

Poštovana koleginice/poštovani kolega,

Ovo je anketa u kojoj dajete svoju ocenu samopouzdanja koje imate prilikom izvođenja stomatoloških procedura čiju ste veštinu izvođenja savladali na prethodnim godinama studija stomatologije.

Za svaku kliničku proceduru treba da date ocenu od 1 do 5 o stepenu samopouzdanja koji imate prilikom njenog izvođenja. Ocene imju sledeće značenje:

1. nemam samopuzdanja prilikom izvođenja ove procedure
2. imam malo samopouzdanja pri izvođenju ove procedure
3. imam umereno samopuzdanje pri izvođenju ove procedure
4. imam dosta samopouzdanja pri izvođenju ove procedure
5. potpuno sam samouveren pri izvođenju ove procedure

Za davanje odgovora na pitanja u okviru ankete Vam neće biti potrebno više od 15 minuta.

| Stomatološka procedura | Ocena samopouzdanja |
| --- | --- |
| 1.          Pregled pacijenta, anamneza, primena dijagnostičkih testova |  |
| 2.          Vođenje medicinske dokumentacije |  |
| 3.          Dijagnostika karijesa |  |
| 4.          Terapija karijesa; Preparacija kaviteta |  |
| 5.          Priprema i postavka materijala za ispune |  |
| 6.          Dijagnostika oboljenja pulpe |  |
| 7.          Dijagnostika oboljenja apeksnog parodoncijuma |  |
| 8.          Endodontska terapija inficiranih i neinficiranih kanala korena (faze rada) |  |
| 9.          Endodontska terapija inficiranih i neinficiranih kanala korena (medikacija kanala) |  |
| 10.      Endodontska terapija inficiranih i neinficiranih kanala korena (definitivna opturacija kanala) |  |
| 11.      Izrada anatomskog i situacionih otiska kod bezubih i krezubih pacijenata |  |
| 12.      Izrada funkcionalnih otisaka za parcijalne i totalne proteze |  |
| 13.      Određivanje MVO pri izradi različitih zubnih nadoknada |  |
| 14.      Proba postave zuba kod različitih oblika mobilnih zubnih nadokanada |  |
| 15.      Predaja gotovih različitih oblika mobilnih zubnih nadoknada |  |
| 16.      Preparacija kanala korena i izrada modela livene nadogradnje |  |
| 17.      Preparacija zuba za različite zubne nadoknade |  |
| 18.      Otiskivanje preparisanih zuba. Izrada privremenih kruna |  |
| 19.      Proba metalne konstrukcije metalokeramičkih nadoknada. Proba keramike. Reokludacija |  |
| 20.      Cementiranje fiksnih nadoknada |  |
| 21.      Dijagnostika oboljenja parodoncijuma i oralne sluzokože |  |
| 22.      Eliminacija zapaljenja parodoncijuma; Kauzalna terapija obolelog parodoncijuma |  |
| 23.      Dijagnostika mukogingivalnih anomalija |  |
| 24.      Korekcija urođenih, stečenih ili razvojnih mukogingivalnih anomalija |  |
| 25.      Eliminacija parodontalnih džepova hirurškim putem |  |
| 26.      Dijagnostika traumatske okluzije; Eliminacija traumatskog kontakta selektivnim brušenjem |  |
| 27.      Procena uspeha kauzalne terapije parodontopatije |  |
| 28.      Prepoznavanje faktora rizika |  |
| 29.      Uzimanje brisa; Izrada terapijskog plana |  |
| 30.      Eliminacija lokalnih iritacija; Lokalna aplikacija leka |  |
| 31.      Aplikovanje terminalnihih i sprovodnih anestezija u usnoj duplji.Dijagnostika, prevencija i terapija komplikacija lokalne anestezije |  |
| 32.      Vađenje izniklih zuba i zbrinjavanje rane posle vađenja zuba. Dijagnostika i prevencija komplikacija u toku i posle vađenja zuba |  |
| 33.      Terapija komplikacija u toku i posle vađenja zuba. Komplikovano vađenja zuba (separacija) |  |
| 34.      Hirurško vađenje zalomljenih korenova i delimično izniklih zuba. |  |
| 35.      Postavljanje indikacija i kontraindikacija za vađenje neizniklih (impaktiranih) zuba |  |
| 36.      Dijagnostika i terapija akutnih i hroničnih dentogenih infekcija |  |
| 37.      Dijagnostika pacijenata rizika i upoznavanje sa njihovom pripremom za oralnohirurške zahvate. Vađenje zuba i manji oralnohirurški zahvati kod pacijenata rizika uz odgovarajuću pripremu ovih pacijenata |  |
| 38.      Uspostavljanje lokalne hemostaze u toku, kao i posle vađenja zuba i manjih oralnohirurških intervencija. |  |
| 39.      Dijagnostika oroantralnih komunikacija i fistula.Konzervativno zbrinjavanje oroantralnih komunikacija |  |
| 40.      Dijagnostika hroničnih periapikalnih lezija i viličnih cista.Postavljanje indikacija za hirurško lečenje hroničnih periapikalnih lezija i viličnih cista |  |

Hvala na učestvovanju u studiji!
