# Supplementary material for: The effect of COVID-19 pandemic on final year dental students’ self-confidence level in performing clinical procedures
Source: PLoS One. 2021 Oct 14;16(10):e0257359. doi: 10.1371/journal.pone.0257359 (PMC8516192; doi:10.1371/journal.pone.0257359)
Supplement: S2 Appendix — (DOCX) [file pone.0257359.s004.docx]

**Informed consent for the participants**

Dear colleagues,

You belong to the generation of dental students who received a part of education in specific conditions of COVID-19 Pandemic. We believe this influenced not only the way of your knowledge acquisition, but also the level of your self confidence when performing dental procedures. Therefore, we designed this research in order to gather and analyze your attitudes on our own self confidence in performing dental procedures you should be trained to conduct after the completion of previous courses and exams. The results of this study could help in teaching reformulation and also estimation of your future educational needs. We would be grateful if you would participate in the study by responding to the questionnaire (in Serbian) you may access [here](https://docs.google.com/forms/d/e/1FAIpQLSekeCKyYpNYDHj5olAufk-alznyhuC3kOhPnIxo2gx_CTn7VA/viewform?vc=0&c=0&w=1&flr=0). By completing the on-line questionnaire it will be considered that you have given the written consent for participating and utilization of the gained data for statistical analyses within the current investigation. The participation is completely voluntary and your decision to participate or not, as well as the given answers would have no impact on your current studying status at the Dental School.

On the behalf of research team,

Dr. Aleksandra Milić Lemić, associate professor

Dr. Jugoslav Ilić, associate professor.

**Questionnaire**

Dear colleague,

This is the questionnaire in which you may give the estimation of your own self confidence in performing dental procedures as the skills you have gained in previous study years.

You should grade self confidence in each given dental procedure by giving one of the following grades:

1. I don’t have self confidence while performing this procedure at all
2. I have little self confidence while performing this procedure
3. I have moderate self confidence while performing this procedure
4. I have self confidence while performing this procedure
5. I am completely self confident while performing this procedure

You will need no more than 15 min. to complete this questionnaire.

| Dental procedure | Self-confidence score |
| --- | --- |
| 1. Clinical examination in restorative dentistry |  |
| 1. Maintaining of dental records in restorative dentistry |  |
| 1. Caries diagnostics and planning of therapy |  |
| 1. Cavity preparation |  |
| 1. Placement of all types of tooth restorations |  |
| 1. Pulpal diagnosis |  |
| 1. Diagnosis of apical periodontitis |  |
| 1. Endodontic therapy- root canal preparation |  |
| 1. Endodontic therapy-irrigation and medication |  |
| 1. Endodontic therapy- root canal obturation |  |
| 1. Anatomic and situational impressions |  |
| 1. Functional impression for removables and complete dentures |  |
| 1. Establishing of intermaxillary relationship |  |
| 1. Teeth set up try in |  |
| 1. Delivery of dentures |  |
| 1. Root canal preparation for casted post |  |
| 1. Tooth preparation in fixed prosthetics |  |
| 1. Impressions in fixed prosthetics |  |
| 1. Try-in of ceramic fused to metal reconstructions. Rearticulation |  |
| 1. Cementing of fixed prosthetic |  |
| 1. Diagnosis of periodontal pathology |  |
| 1. Causal therapy of periodontal disease |  |
| 1. Diagnosis of mucogingival anomalies |  |
| 1. Correction of micogingival anomalies. |  |
| 1. Surgical elimination of periodontal pockets. |  |
| 1. Diagnosis and elimination of traumatic dental contacts |  |
| 1. Evaluation of causal therapy success |  |
| 1. Recognition of risk factors for periodontal disease |  |
| 1. Swab taking. |  |
| 1. Elimination of local irritations. Local medication |  |
| 1. Administering of local anesthesia in oral tissues. |  |
| 1. Extraction of erupted teeth and wound management. |  |
| 1. Tooth extractions with separation of roots. |  |
| 1. Surgical extractions of fractured radices |  |
| 1. Recognition of indications for extraction of impacted teeth. |  |
| 1. Diagnosis and therapy of acute and chronic dental infections |  |
| 1. Tooth extraction and minor oral surgery in medically compromised patients. |  |
| 1. Haemostasis in tooth extractions and minor oral surgery |  |
| 1. Diagnosis and conservative therapy of oroantral communications and fistulas. |  |
| 1. Diagnosis of periapical lesions and cysts in jaws. |  |

Thank you for the participation!
